# Influence of CD8+ T regulatory cells on intraocular tumor development

**DOI:** 10.3389/fimmu.2012.00303

**Published:** 2012-09-28

**Authors:** Kyle C. McKenna, Dana M. Previte

**Affiliations:** Departments of Ophthalmology and Immunology/Medicine, University of Pittsburgh, University of Pittsburgh Cancer InstitutePittsburgh, PA, USA

**Keywords:** eye, tumor, CD8, Treg, CTL, immunosuppression, ACAID, immune evasion

## Abstract

The interior of the eye, or uvea, is a site of immune privilege where certain immune responses are attenuated or completely excluded to protect non-regenerating tissues essential for vision. One consequence of this immunoregulation is compromised immune mediated elimination of intraocular tumors. For example, certain murine tumor cell lines which are rejected by host immune responses when transplanted in the skin grow progressively when placed in the anterior chamber (a.c.) of the eye. Progressive ocular tumor growth occurs despite induction of tumor-specific CD8+ T cell responses capable of eliminating a subsequent tumor challenge in the skin or opposite eye. Why these CD8+ T effectors fail to eliminate established ocular tumors is not known. It is well appreciated that growth of tumors in the a.c. induces the generation of immunosuppressive CD8+ T regulatory (Treg) cells. However, the contribution of CD8+ Treg in ocular tumor progression remains unclear. Several studies indicate that these CD8+ Treg target responding CD4+ T cells to inhibit their induction of macrophage-dependent delayed type hypersensitivity (DTH) responses to tumor antigens (Ags). However, induction of tumor-specific CD4+ T cell responses does not assure intraocular tumor elimination. This review is focused on how CD8+ Treg could influence the tumoricidal activity of ocular tumor-specific CD8+ T effector cells.

## Introduction

**Figure 1:**
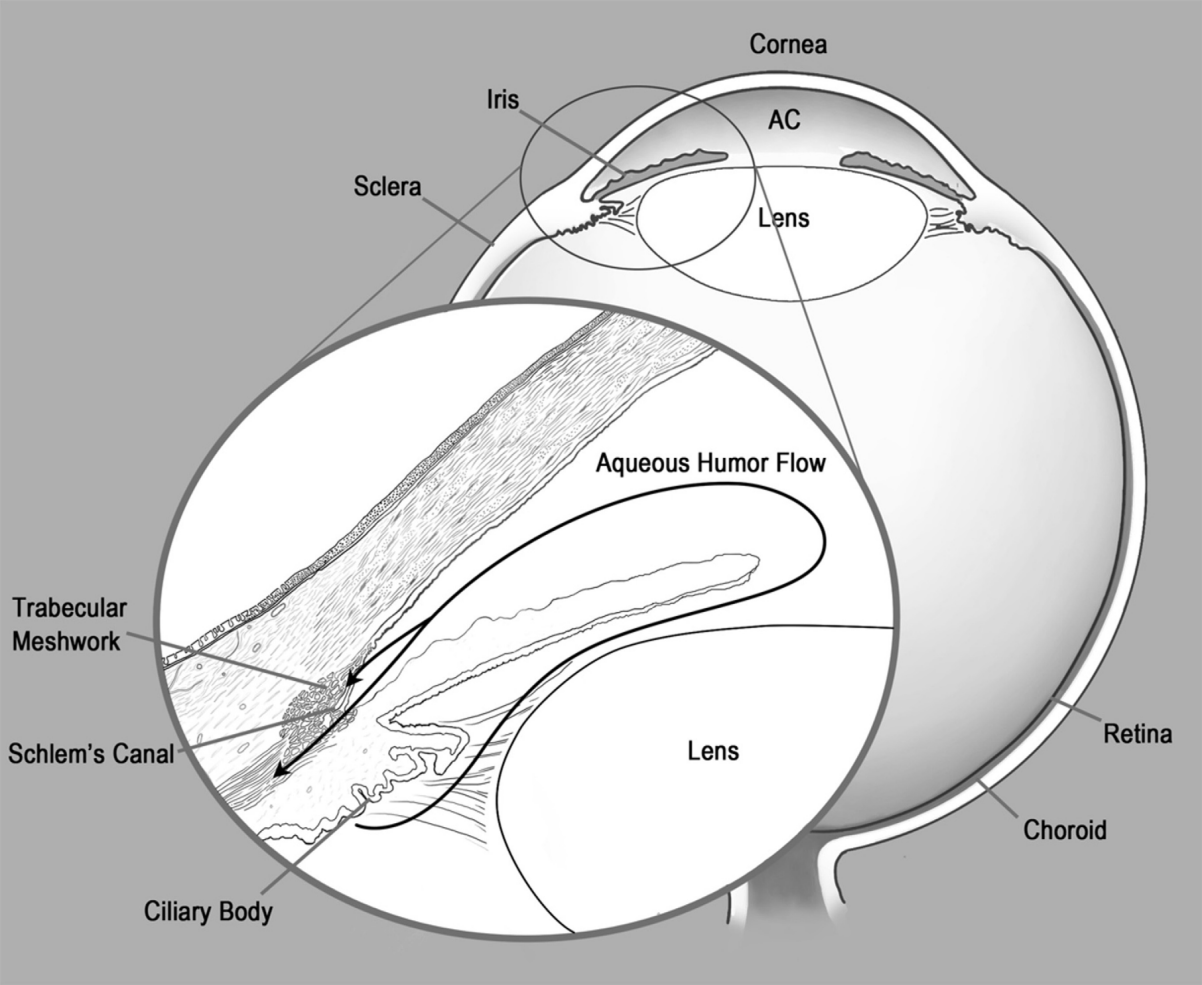
**Ocular anatomy and drainage.** Anterior chamber (AC).

The concept of immune privilege was first advanced in the 1940s by the Nobel laureate Sir Peter Medawar. While studying tissue transplantation he observed that foreign skin grafts which were normally rejected when transplanted subcutaneously (s.c.), persisted sometimes indefinitely when transplanted into other sites that he termed “immune privileged” (Medawar, 1948). One immune privileged site was the anterior chamber (a.c.) of the eye, the aqueous humor (AqH) filled cavity located directly below the cornea and above the lens (Figure 1). As the a.c. is separated by a blood–AqH barrier and lacks demonstrable afferent lymphatic drainage, ocular immune privilege was originally explained by sequestration of ocular antigens (Ags) from the circulating immune system. However, seminal findings by Kaplan and Streilein in 1977 which demonstrated that antibody responses were generated to foreign Ags placed in the a.c. (Kaplan and Streilein, 1977) clearly showed that the immune system was not ignorant of ocular Ags. We now know that certain immune responses are attenuated or completely excluded from the eye to protect non-regenerating ocular tissues essential for vision. This ocular immune privilege is maintained by unique anatomical and biochemical features of the eye along with the generation of systemic tolerance to ocular Ags which is mediated by regulatory T cells (Treg). In this review we focus on how CD8+ Treg generated during intraocular tumor growth could influence the tumoricidal activity of tumor-specific CD8+ T effector cells.

## Mechanisms of ocular immune privilege

### Influence of ocular anatomy on immune privilege

The absence of afferent lymphatics (Bill, 1977), an avascular cornea (Patel and Dana, 2009), and tight junctions between vascular endothelial cells in the iris and retina (Crane and Liversidge, 2008) are barriers to the generation and expression of ocular immune responses. However, these barriers are not absolute as administration of soluble Ags into the a.c. has been shown to induce Ag-specific CD8+ (McKenna et al., 2002, 2005) and CD4+ (Egan et al., 1996; Perez et al., 2000) T cell expansion in the ipsilateral submandibular lymph nodes (LNs) and spleens of mice. In addition, activated T cells can enter even a non-inflamed retina to induce uveitis (Xu et al., 2003). Therefore, ocular anatomy may increase the threshold for the generation and expression of ocular immune responses but clearly does not prevent them.

Ags encountered within the a.c are thought to exit the eye via the normal drainage of AqH (Bill, 1977) (Figure 1 inset). AqH is continually generated by the ciliary body, fills the a.c., and then primarily drains via the trabecular meshwork and Schlem's canal directly into the blood stream. A much smaller percentage of AqH travels by uveal-scleral flow into the cilary muscle and then traverses the choroid and sclera to be drained by conjunctival lymphatics (Bill, 1977). As we will discuss in subsequent sections, the spleen is essential for the generation of Treg which also contribute to maintaining ocular immune privilege. Therefore, preferential trafficking of ocular Ags via the bloodstream to the spleen may favor the induction of T cell tolerance. In addition, antigen presenting cells (APCs) from the iris and ciliary body have been shown to selectively traffic to the spleen (Wilbanks and Streilein, 1992).

### Biochemical barriers to ocular immune responses

**Figure 2:**
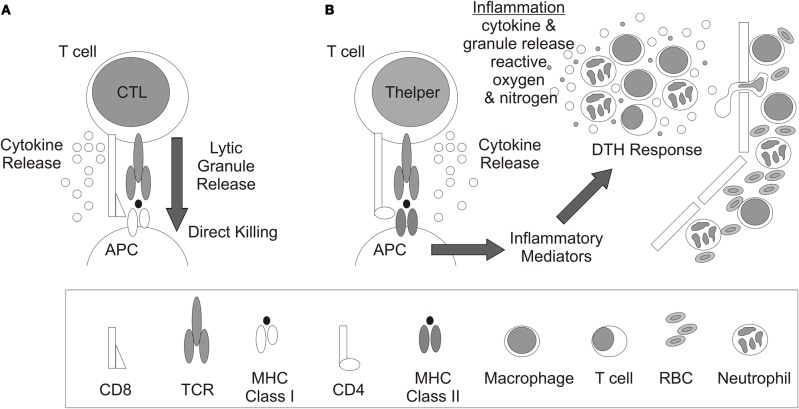
**T cell recognition and response.** Antigen recognition and response by **(A)** CD8+ cytolytic T lymphocytes (CTL) and **(B)** CD4+ T helper cells. Antigen presenting cell (APC), cluster of differentiation (CD), delayed type hypersensitivity response (DTH), T cell receptor (TCR), major histocompatibility complex (MHC), and red blood cell (RBC).

T cells recognize processed peptides presented on major histocompatibility complex (MHC) molecules. In general, peptides presented by MHC Class I molecules are recognized by CD8+ T cells whereas CD4+ T cells recognize peptides complexed with MHC Class II molecules (Figures 2A,B). CD8+ T cells differentiate into cytotoxic T lymphocytes (CTL) which primarily eliminate infected or malignant cells by release of lytic granules although cytokines are also released (Figure 2A) whereas CD4+ Thelper cells primarily release cytokines to influence other immune cells. For example, during DTH responses, CD4+ T cells express IFNγ and/or IL-17 which recruits and activates macrophages and neutrophils to promote inflammation (Figure 2B). The response is delayed due to the requisite time for Ag-specific T cells to expand in draining LN and then migrate to the site of Ag exposure.

Although the majority of cells within the body express MHC Class I molecules and can be induced to express MHC Class II molecules by stimulation with interferon gamma (IFNγ), ocular tissues demonstrate atypical expression of MHC Class I and II. For example, corneal endothelial cells express very low levels of MHC Class I which protects these non-regenerating cells from lysis by CD8+ CTL (Abi-Hanna et al., 1988). Ocular melanocytes are impaired in expression of MHC Class II which may mitigate CD4+ T cell mediated inflammation (Radosevich et al., 2004, 2007).

AqH contains soluble immune suppressive molecules including cytokines, neuropeptides, and growth factors that have been shown to inhibit adaptive immune responses. Benezra and Sacks documented over forty years ago that AqH could inhibit proliferation of naïve T cells following PHA stimulation (Benezra and Sachs, 1974), and we now know that high concentrations of the cytokine TGFβ2 in AqH contributed to this suppression (Kaiser et al., 1989; Cousins et al., 1991). TGFβ2 is normally present in a latent form within the AqH and elegant work by Masli and coworkers demonstrated a critical role of thrombospondin-1 in activation of latent TGFβ2 to preserve ocular immune privilege (Masli et al., 2006).

Taylor and coworkers have clearly shown that AqH directs *in vitro* primed CD4+ T cells away from an IFNγ expressing phenotype and toward a TGFβ1 producing Treg type (Taylor et al., 1997). Factors within AqH including TGFβ2 and alpha-melanocyte stimulating hormone (α-MSH) can alone generate Treg (Nishida and Taylor, 1999). In combination, α-MSH increases the frequency of Treg by abrogating the anti-proliferative effects of TGFβ (Nishida and Taylor, 1999). Additional factors within AqH that favor Treg generation include somatostatin which induces α-MSH production in T cells (Taylor and Yee, 2003) and vasoactive intestinal peptide (VIP) which inhibits IFNγ production in effector T cells (Taylor et al., 1994).

AqH also contains molecules that inhibit innate immune responses. For example, high concentrations of ascorbic acid in AqH have been shown to inhibit myeloperoxidase activity of neutrophils (Rosenbaum et al., 1985) and macrophage migration inhibitory factor (MIF) in AqH inhibits NK cell activity (Apte et al., 1998). AqH-mediated changes to the innate immune response also influence the adaptive immune response. For example, pretreatment of macrophages with TGFβ2 decreased their expression of CD40 and IL-12 and increased TGFβ1 expression (Takeuchi et al., 1998). Consequently, CD4+ T cells stimulated with TGFβ2 treated APC were deviated from IFNγ producing T cells to TGFβ1 producing Treg (Takeuchi et al., 1998; Keino et al., 2006b).

The interior of the eye is also lined by a continuous layer of pigmented epithelial (PE) cells of the iris, ciliary body, and retina along with the corneal endothelium. All of these ocular tissues have been shown to induce T cells to become immunosuppressive Treg *in vitro* (Sugita et al., 2006, 2008, 2009, 2011). Iris PE cells best convert CD8+ T cells into Treg via their expression of CD86 which engages CTLA-4 on activated CD8+ T cells (Sugita et al., 2008). In contrast, retinal PE cells and corneal endothelial cells express CTLA-2α which better converts CD4+ T cells into Tregs by decreasing cathepsin-L activity in T cells (Sugita et al., 2008, 2009). Expression of TGFβ1 by CD8+ (Sugita et al., 2008) and CD4+ Tregs (Sugita et al., 2006) contributes to their immunosuppressive activity. In addition, CD8+ Tregs generated by iris PE express CD86 (Sugita et al., 2006) and the immnosuppressive molecule programmed death 1 (PD-1) to suppress CTLA-4+ and PD-1 ligand + T cell effectors (Sugita et al., 2010).

Cells lining the interior of the eye also express death inducing molecules including CD95/FasL (Griffith et al., 1995a), and PD-1 ligand (Hori et al., 2006) which can induce apoptosis of effector T cells expressing CD95/Fas and PD-1 respectively. The significance of these death-inducing molecules is very apparent in corneal transplantation as corneal allografts deficient in CD95/FasL (Stuart et al., 1997; Yamagami et al., 1997) or PD-1 ligand (Hori et al., 2006) are rejected with increased frequency. Apoptotic CD4+ T cells were observed at the allograft junction of accepted corneas whereas CD4+ T cells accumulated in rejecting corneal allografts (Hori et al., 2006) which supported a death inducing mechanism for these molecules.

**Figure 3:**
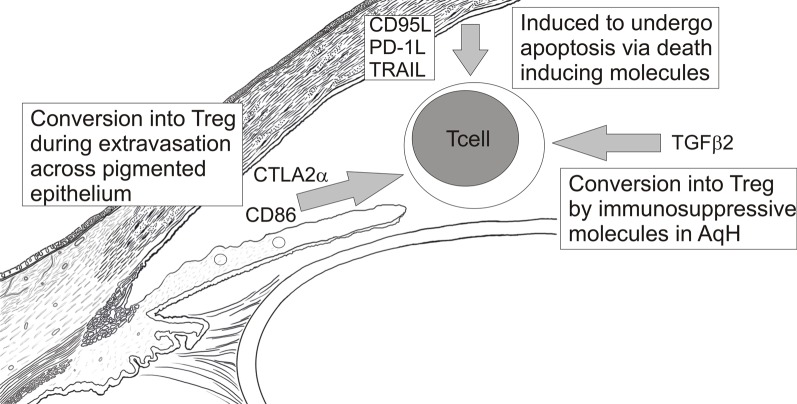
**Mechanisms of T cell inactivation within the anterior chamber**.

Taken together, the above data suggest a model (Figure 3) in which activated effector T cells are inactivated by their conversion into Treg as they extravasate from blood vessels and cross epithelial boundaries to enter the eye. Those effectors that escape this regulation may be converted into Treg by immunosuppressive factors within ocular fluids, like AqH, or induced to undergo apoptosis by death inducing molecules within the eye. Again, these barriers to ocular immune responses are not absolute as intravenous transfer of activated effector CD4+ T cells specific for ocular Ags can induce either anterior (Lai et al., 1999) or poster uveitis (Xu et al., 2003). Similarly, Zhou et al. (2012) recently showed that effector T cells specific for a retinal Ag did not become Treg when injected into the posterior chamber of the eye (Zhou et al., 2012). In addition, naïve mice are protected from an ocular tumor challenge if first infused intravenously with tumor-specific CD8+ T effectors (Niederkorn and Streilein, 1984). These data clearly indicate that activated effectors can overcome immunosuppressive mechanisms within the eye. Hence, these biochemical barriers must primarily raise the threshold of expression of ocular immune responses.

### Anterior chamber associated immune deviation (ACAID)

One consequence of stringent control of ocular immune responses is compromised immune mediated elimination of intraocular tumors. For example, certain murine tumor cell lines (Table 1) that were rejected when transplanted in the skin grew progressively when placed in the a.c. of the eye. Progressive growth of these intraocular tumors was not due to a failure to prime immune responses. Rather, CD8+ CTL (Niederkorn and Streilein, 1983a; Ksander and Streilein, 1989) and cytotoxic antibody responses (Niederkorn and Streilein, 1982a) specific for tumor Ags were equivalent or greater than those observed in mice that rejected the same tumors in the skin. However, ocular tumor growth also generated immunosuppressive CD8+ Treg that inhibited CD4+ T cell mediated DTH responses to tumor Ags (Streilein and Niederkorn, 1985). This unique immune response which was deviated from the conventional immune response observed when tumors were injected in the skin was described by the general term a.c. associated immune deviation or ACAID.

**Table 1:** **Transplantable intraocular tumor models**.

**Tumor cell line**	**Tumor origin**	**Recipient mouse strain**	**Antigens on tumor**	**Intraocular tumor growth**	**Skin tumor tumor growth**	**References**
P815	DBA/2	Balb/C	Minor MHC	Progressive	Rejection	Niederkorn et al., 1981
P815	DBA/2	C57Bl/6	Minor + Major MHC	Rejection	Rejection	Niederkorn et al., 1981
B16F10	C57Bl/6	A/J	Minor + Tumor Ag	Progressive	Rejection	Niederkorn and Streilein, 1983b
B16F10	C57Bl/6	C57Bl/6	Tumor Ag	Progressive	Progressive	Niederkorn, 1984
E.G7-OVA	C57Bl/6	C57Bl/6	Ovalbumin	Progressive	Rejection	McKenna and Kapp, 2006
Ad5E1	C57Bl/6	C57Bl/6	Adenovirus	Rejection	Rejection	Schurmans et al., 2001
UV-5C25	C57Bl/6	C57Bl/6	Tumor Ag	Rejection	Rejection	Knisely et al., 1987
P91	DBA/2	DBA/2	Tumor Ag	Rejection	Rejection	Knisely et al., 1987

**Figure 4:**
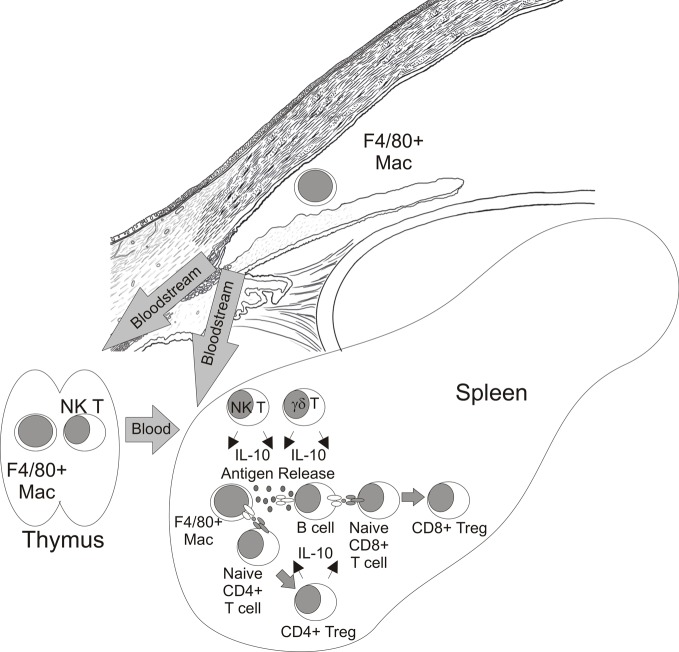
**Paradigm of CD8+ Treg generation in ACAID**.

ACAID has been primarily defined by suppression of DTH responses to Ags that were first encountered in the a.c. and has been demonstrated using tumors (Niederkorn et al., 1981), haptenated splenocytes (Waldrep and Kaplan, 1983), viruses (Ksander and Hendricks, 1987), and soluble Ags (Wilbanks and Streilein, 1990). The induction of ACAID is a complicated process which requires several tissues including the eye (Niederkorn and Streilein, 1982b), spleen (Streilein and Niederkorn, 1981), thymus (Wang et al., 1997), and sympathetic nervous system (Li et al., 2004; Vega et al., 2009). The current paradigm for the induction of ACAID (Figure 4) suggests that ocular Ags are processed by F4/80+ macrophages that were influenced by TGFβ2 in AqH (Wilbanks et al., 1991, 1992; Wilbanks and Streilein, 1991). These APCs travel from the eye via the bloodstream to the thymus and marginal zone of the spleen. Within the thymus CD4− CD8− NKT cells are generated (Wang et al., 2001) which migrate to the marginal zone of the spleen via a MIP-2 chemokine gradient created by F4/80+ macrophages from the eye (Faunce et al., 2001; Faunce and Stein-Streilein, 2002). These F4/80+ macrophages present Ags directly as well as release Ag which is internalized, processed and presented by B cells (D'Orazio et al., 2001). Coordinate interactions between F4/80+ macrophages (Lin et al., 2005), B cells (D'Orazio et al., 2001), NKT cells (Sonoda et al., 1999; Sonoda and Stein-Streilein, 2002), and γδ T cells (Skelsey et al., 2001; Xu and Kapp, 2002) along with production of IL-10 (D'Orazio and Niederkorn, 1998; Sonoda et al., 2001; Ashour and Niederkorn, 2006) culminates in the generation of CD4+ and CD8+ Treg which suppress the induction or expression of DTH responses respectively (Streilein and Niederkorn, 1985; Wilbanks and Streilein, 1990).

The significance of ACAID in ocular immune privilege is clearly demonstrated in splenectomized mice in which ACAID is terminated (Streilein and Niederkorn, 1981). Splenectomy prevents the generation of CD4+ and CD8+ Tregs, restores DTH responses to ocular Ags, and promotes spontaneous rejection of immunogenic tumors placed in the a.c. which would otherwise grow progressively (Streilein and Niederkorn, 1981). Corneal allografts are also rejected with greater frequency in splenectomized mice (Yamagami and Dana, 2001).

### CD8+ Treg

T cells function to both eliminate pathogens and to resolve immune responses which, if left uncontrolled, would cause immunopathology and potentially autoimmunity. The seminal experiments by Sakaguchi in 1995 identified that immunosuppressive CD4+ CD25(IL-2Rα)+ T cells protected mice from autoimmunity mediated by CD4+ CD25– T cells (Sakaguchi et al., 1995). These CD4+ Treg represented a distinct lineage generated in the thymus which could be defined by expression of the transcription factor forkhead box P3 (FoxP3) (Fontenot et al., 2003). In addition to these “natural Treg,” peripheral CD4+ T cells could also be induced to express FoxP3 and demonstrated regulatory activity although FoxP3 negative CD4+ T cells have also been shown to be immunosuppressive (Shevach, 2009).

It is important to note that immunosuppressive activity of T cells was first described twenty years earlier by Gershon and Kondo (Gershon and Kondo, 1971) who demonstrated that T cells could transfer Ag-specific tolerance to naïve mice. However, their “infectious tolerance” model was mediated by CD8+ T cells. Like CD4+ Treg, both natural and adaptive CD8+ Treg have been described. For example, transfer of natural CD122(common γ-chain receptor)+ CD8+ Treg prevented autoimmunity normally observed in CD122 deficient mice (Rifa'i et al., 2004). In addition, certain treatments (Glatiamer acetate and Fc-ILT3) which mitigate autoimmune conditions are associated with an expansion of induced CD8+ Treg cells (Tennakoon et al., 2006; Vlad et al., 2008). With the exception of FoxP3, there are no well defined markers that distinguish CD8+ T effector cells from CD8+ Treg cells.

## Immune responses to intraocular tumors

As mentioned previously, certain tumors transplanted in the a.c. of the eye grow progressively despite induction of tumor-specific CTL (Niederkorn and Streilein, 1983a; Ksander and Streilein, 1989). A simple explanation for this phenomenon is that CD8+ CTL do not accumulate within the eye, either because they fail to infiltrate ocular tumors or because they undergo apoptosis within the eye due to ocular expression of death inducing molecules (Griffith et al., 1995a; Yamagami et al., 1997; Hori et al., 2006). However, this is clearly not the case as primary uveal melanomas are often infiltrated by CD8+ T cells (de la Cruz et al., 1990; Durie et al., 1990; Meecham et al., 1992; Ksander et al., 1998; McKenna et al., 2009). Similarly, CD8+ T cells accumulated within progressively growing tumors transplanted in the a.c. of mice (Ksander et al., 1991; Vicetti Miguel et al., 2010), and T cells isolated from spleens of mice primed to tumor Ags protected naïve mice from an ocular tumor challenge when transferred intravenously (Niederkorn and Streilein, 1984). As transferred T cells influenced tumor numbers within the a.c., these data also indicated that T cells could exert their tumoricidal effector function within the immune privileged eye at least in some circumstances. These data also argue against a conversion of CD8+ CTL into CD8+ Treg within the eye.

Progressive growth of tumors in the a.c. fails to generate CD4+ T cell dependent DTH responses to ocular tumor Ags (Niederkorn and Streilein, 1983a; Streilein and Niederkorn, 1985), and restoration of these DTH responses has been associated with rejection of intraocular tumors. For example, splenectomized Balb/C mice spontaneously eliminated P815 tumors placed in the a.c. and DTH responses to tumor Ags were restored (Streilein and Niederkorn, 1981). Similarly, P91 tumors (a P815 variant) induced strong DTH responses when transplanted in the a.c. of syngeneic DBA/2 mice and these intraocular tumors were also rejected (Niederkorn and Meunier, 1985). The immunopathological features of intraocular P91 tumor rejection resembled a DTH response as there was neutrophil infiltration, extensive damage to normal ocular tissues (including destruction of the microvasculature), and ischemic bulk necrosis (Knisely et al., 1987) resulting in ocular atrophy, termed phthsis. While these data suggested that DTH responses were critical for ocular tumor elimination, additional experiments showed that administration of anti-CD4 antibodies, which abrogated DTH responses measured in the footpad, did not prevent rejection of intraocular P91 tumors (Niederkorn et al., 1990). Similarly, we have observed that phthsical rejection of P815 tumors in splenectomized Balb/C mice was not influenced by CD4+ T cell depletion (our unpublished observations). In contrast, CD8 T cell depletion resulted in progressive growth of P815 (our unpublished observation and P91 tumors in the a.c. of DBA/2 mice (Niederkorn et al., 1990) although strong P91-specific DTH responses were observed in the footpad (Niederkorn et al., 1990). These data indicated that CD4+ T cell mediated DTH responses to tumor Ags were excluded from the eye. More importantly, CD8+ and not CD4+ T cells were most critical for elimination of intraocular P91, or P815 tumors by inducing expression of a “DTH like” immune response within the eye.

The immune suppressive mechanisms that normally exclude CD4+ T cell mediated DTH responses from the eye have not been defined. However, CD4+ T cell infiltration of ocular tumors does not appear to be compromised as Ad5E1 tumors are spontaneously rejected when placed in the a.c. by a process that requires CD4+ T cells, macrophages, and IFNγ (Schurmans et al., 2001; Wang et al., 2003; Boonman et al., 2006; Dace et al., 2008). Rejection of these ocular tumors is due to direct effects of IFNγ on tumors as Ad5E1 were also rejected in IFNγ receptor 1 (IFNγR1) deficient mice (Dace et al., 2007). As Ad5E1 tumors do not express MHC Class II these data suggest a model in which CD4+ T cells infiltrating ocular tumors express IFNγ only after engaging tumor Ags complexed with MHC Class II that are presented by ocular APC, most probably intratumoral macrophages. The direct effects of IFNγ on Ad5E1 tumors include inhibiting proliferation, and inducing expression of the death inducing molecule TRAIL-R2 and several anti-angiogenic molecules which in combination leads to non-phthsical ocular tumor elimination (Wang et al., 2003; Dace et al., 2007, 2008). Interestingly, Coursey and coworkers recently identified an Ad5E1 variant (Clone 2.1) that was rejected in a phthsical manner (Coursey et al., 2011). Destructive rejection of these tumors required CD4+ T cells and nitric oxide producing macrophages. TNFα expression was critical for inducing Ad5E1 tumor death in a manner which promoted differentiation of tumoricidal macrophages.

### Influence of Treg cells on intraocular tumor growth

Administration of tumors into the a.c. of the eye induces CD8+ Tregs which transfer suppression of DTH responses to recipient mice (Streilein and Niederkorn, 1985). These CD8+ Treg are not generated in splenectomized mice that reject tumors transplanted in the a.c. so it is tempting to speculate that they contribute to progressive intraocular tumor growth. However, it is important to note that these Treg do not inhibit the generation of systemic CD8+ CTL or antibody responses directed against ocular tumors (Niederkorn and Streilein, 1983a; Ksander and Streilein, 1989). Therefore, their immunosuppressive effects could only be at the “efferent” stage of the immune response targeting effector immune cells within primary ocular tumors. This notion is somewhat complicated by the observation that mice bearing progressively growing tumors in one eye reject a subsequent tumor challenge in the opposite eye or skin− a phenomenon termed “intracamerally induced concomitant immunity” (Niederkorn and Streilein, 1983b; McKenna and Kapp, 2006). The induction of CD8+ Treg and CD8+ CTL occur with similar kinetics requiring at least 7–10 days to develop (Streilein and Niederkorn, 1981; McKenna and Kapp, 2006). Therefore as the second tumor challenge occurred at a time when CD8+ Treg would have already developed these data indicate that Treg do not influence the tumoricidal activity of immune responses within the microenvironment of the subsequent tumor challenge. However, the requirements for rejection of established primary ocular tumors could be different from those necessary to reject subsequent tumor challenges and thus more or less sensitive to immunoregulation. For example, although Ad5E1 tumors are spontaneously rejected in the a.c. of C57Bl/6 mice by a process that requires CD4+ T cells, macrophages and IFNγ (Schurmans et al., 2001; Wang et al., 2003; Boonman et al., 2006; Dace et al., 2007, 2008), IFNγ deficient mice reject Ad5E1 tumors placed s.c. in the skin and these mice also reject a subsequent Ad5E1 challenge in the a.c. (Dace et al., 2008). Hence, IFNγ is required for rejection of established Ad5E1 ocular tumors but not for protection from a subsequent ocular tumor challenge.

Tumor burden is also significantly greater in established ocular tumors thereby requiring a stronger immune response to promote tumor elimination which should be more sensitive to immunoregulation. For example, serum or LN cells from mice immunized with P815 tumors s.c. protected naïve mice from an ocular tumor challenge if given 7 days before or at the same time as tumor administration in the a.c. but not if given four days after tumor inoculation (Niederkorn and Streilein, 1984). It is important to note that the kinetics and consequences of rejection of intraocular tumors by transferred sera or immune cells were different and significant. Ocular tumors never developed in mice given immune sera and the eye was preserved (Niederkorn and Streilein, 1984) suggesting that cytotoxic antibodies immediately eliminated tumors in the a.c. of the eye. In contrast, mice given immune LN cells developed established intraocular tumors which were then rejected by a process that caused phthsis (Niederkorn and Streilein, 1984). These data suggest that T cell and macrophage dependent DTH responses were involved in tumor elimination. Therefore, CD8+ Treg could target effector T cells or macrophages within ocular tumors to inhibit expression of DTH responses within the eye and as a result promote ocular tumor growth.

### Mechanisms of suppression by CD8+ Treg in ACAID

Characterization of the immune suppressive activity of CD8+ Treg in ACAID has relied entirely on assays that evaluate the inhibition of DTH responses. Specifically, splenocytes from mice given Ag in the a.c. have been shown to inhibit DTH responses when transferred to recipient mice previously immunized with the same Ag (Streilein and Niederkorn, 1985; Wilbanks and Streilein, 1990). In addition, splenocytes from mice given Ag in the a.c. were shown to suppress DTH responses when injected into the skin along with Ag and responder splenocytes from mice immunized with the same Ag in adjuvant. In this “local adoptive transfer assay” (LAT), responder cells alone induced DTH responses and these responses were not suppressed by naïve T cells, or splenocytes from mice given Ag s.c. but only by splenocytes from mice given Ag in the a.c. Further experimentation indicated that the splenic regulatory cell was a CD8+ T Cell (Wilbanks and Streilein, 1990) and this observation has been very reproducible in several different laboratories (Griffith et al., 1996; Nakamura et al., 2003; Cone et al., 2007; Paunicka et al., 2011).

CD8+ Treg isolated from mice given Ag in the a.c. suppress DTH responses in an exquisitely specific manner. For example, splenic CD8+ T cells from mice given bovine serum albumin (BSA) in the a.c. suppress BSA-specific responder cells but not OVA-specific responder cells even if both BSA and OVA Ag are co-injected in the LAT assay to activate BSA-specific CD8+ Treg (Wilbanks and Streilein, 1990). These data clearly argue against “linked suppression” in which CD8+ Treg target APC that express both Ags (Kapp et al., 2006, 2007).

The cellular target of CD8+ Tregs and their mechanism of immune suppression in the DTH response have not been fully elucidated. However, in a related system in which CD8+ Tregs were generated after intravenous injection of TGFβ2-treated Ag-pulsed macrophages, these CD8+ Treg inhibited DTH responses by inducing apoptosis of responder T cells through a Fas/FasL dependent mechanism (Kosiewicz et al., 2004). Similarly, Griffith and coworkers recently demonstrated that CD8+ Treg generated by the administration of HSV-1, or haptenated splenocytes (TNP-SPL) in the a.c. suppressed DTH responses by an apoptotic mechanism involving TRAIL/DR5 interactions (Griffith et al., 2011). In a third system in which CD8+ Treg were generated by injection of haptenated soluble Ag in the a.c., Cone and coworkers also showed that responding T cells were targeted by CD8+ Treg to suppress DTH responses (Cone et al., 2009b). Recognition of responding T cells by CD8+ Treg was restricted by Qa-1 and not MHC Class I (Cone et al., 2009a) which was consistent with the requirement for Qa-1 presentation of Ags by B cells in the generation of CD8+ Treg in ACAID (D'Orazio et al., 2001).

**Figure 5:**
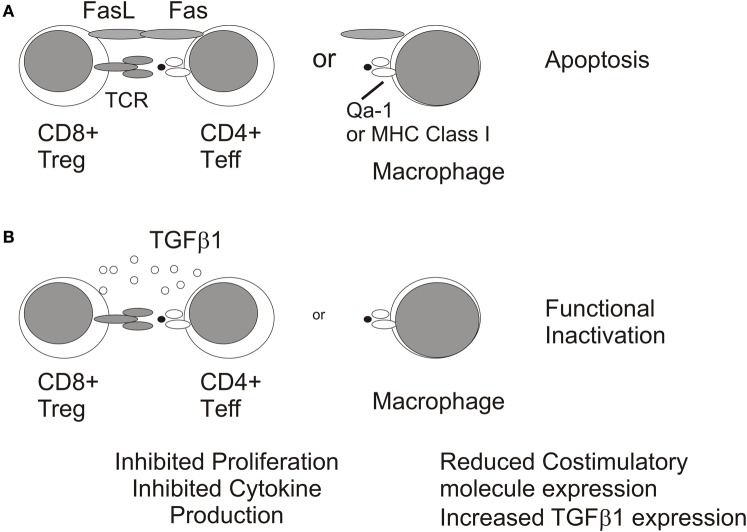
**Mechanisms of immunosuppression by CD8+ Treg.** CD8+ Tregs may inhibit DTH responses by inducing apoptosis of CD4+ T cell effectors or macrophages **(A)** or by functionally inactivating these immune populations via TGFb1 expression **(B)**.

CD8+ Treg that recognize T cell receptor (TCR) peptides presented by Qa-1 have been shown to lyse Vβ3+ CD4+ T cells which normally expand in mice given the superantigen staphylococcal enterotoxin A (SEA) (Hu et al., 2004). These data suggest that under certain circumstances CD8+ Treg are primed to TCR determinants expressed by responding CD4+ T cells. Recognition of a TCR expressed by CD4+ T cells which expanded to Ags administered in the a.c. would nicely explain the specificity of CD8+ Treg in suppression of DTH responses in ACAID. As previously described, ACAID CD8+ Treg only suppress DTH responses to the same Ag as was originally delivered in the a.c. (Wilbanks and Streilein, 1990). Therefore, these data suggest a model in which CD8+ Treg eliminate responding T cell populations by recognizing a unique TCR determinant presented by Qa-1 on T cells (Figure 5A). In support of this model two independent laboratories have identified Ag-specific TCR proteins in serum after administration of Ag in the a.c. which transfer tolerance to naïve recipient mice (Ferguson et al., 1989; Griffith et al., 1995b; Hadjikouti et al., 1995). In addition, CD8+ Treg which suppress OVA-specific DTH responses can be generated by injection of the class II restricted peptide OVA_323−339_ (Kosiewicz and Streilein, 1996) which supports that CD8+ Treg in ACAID are not restricted by MHC Class I and could indicate that CD8+ Treg recognized a TCR determinant expressed by responding OVA_323−339_-specific CD4+ T cells. The inhibition of DTH responses by CD8+ Treg was also associated with absence of immune infiltrates at the site of the LAT assay in the skin which is consistent with elimination of responder T cells (Cone et al., 2009a).

**Figure 6:**
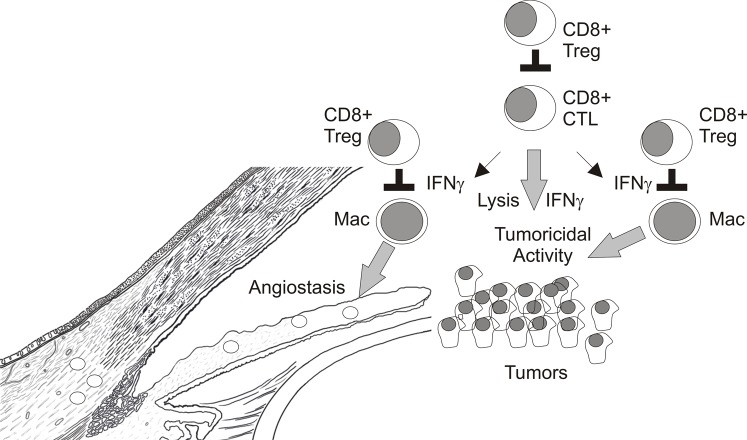
**Potential targets of CD8+ Treg in intraocular tumors**.

A significant caveat to this model is that CD8+ Treg were also generated in CD4+ deficient MHC Class II−/− mice given soluble Ag in the a.c. (Nakamura et al., 2003). Therefore, CD8+ Treg with specificities other than TCR expressed by CD4+ T cells may also be generated. For example, CD8+ OT-I TCR transgenic T cells which recognize OVA peptide 257–264 complexed with Class I K^b^ were induced to become non-lytic CD103+ CD8+ Treg when stimulated with OVA-pulsed-TGFβ2 treated macrophages (Kezuka and Streilein, 2000; Keino et al., 2006a). These OT-I Treg were similar to those observed in ACAID in that they suppressed DTH responses in a LAT assay (Kezuka and Streilein, 2000; Keino et al., 2006a). However, their cellular target may be APC (macrophages and/or dendritic cells) and not responder CD4+ T cells (Figure 5B) as Kapp and coworkers showed in a similar system that OT-I Treg reduced expression of the costimulatory molecule, CD86, on dendritic cells (Kapp et al., 2006). TGFβ has been shown to reduce costimulatory molecule expression on APC (Takeuchi et al., 1998) and is required for the suppressive activity of CD8+ Treg in ACAID (Cone et al., 2009b; Jiang et al., 2009) which could suggest additional non-lytic mechanism for inhibiting DTH responses. However, the suppression of immune responses by non-lytic OT-I CD8+ Treg does not require TGFβ (Kezuka and Streilein, 2000; Kapp et al., 2006) indicating that other immunosuppressive mediators may be involved. It is important to note that the potential contribution of CD8+ Treg in promoting ocular tumor growth would require their recognition of non-CD4+ T cells because CD4+ T cells do not contribute to elimination of intraocular P91 or P815 tumors (Niederkorn et al., 1990 and our unpublished observations). Therefore, CD8+ CTL effectors or intratumoral macrophages are logical targets of CD8+ Treg in the ocular tumor microenvironment (Figure 6).

### Indirect tumoricidal activity of CD8+ T cells

CD8+ T cells play a critical role in immunosurveillance of tumors by recognition of processed peptides presented by MHC Class I molecules on the tumor cell surface. Through release of cytotoxic granules containing granzyme B and perforin CD8+ CTL directly lyse tumor cells (Simon et al., 1997). In addition, CD8+ CTL express IFNγ which can induce apoptosis of certain tumors (Wall et al., 2003) and/or influence hematopoietic and non-hematopoietic cells within the tumor microenvironment to affect tumor growth (Blankenstein, 2005). For example, IFNγ dependent regression of B16-OVA melanomas transplanted in the skin by CD8+ OVA-specific OT-I T cell effectors required IFNγR1 expression by host cells (Schuler and Blankenstein, 2003). An inhibition of tumor angiogenesis preceded tumor regression in other models of IFNγ mediated rejection of tumors by CD8+ T cells suggesting that IFNγ targeted vascular endothelial cells, fibroblasts, and/or pro-angiogenic macrophages (Qin et al., 2003).

IFNγ may also promote tumoricidal activity in other immune cell populations within the tumor microenvironment. For example, regression of established E.G7-OVA skin tumors by CD8+ OT-I CTL effectors required IFNγ but not perforin expression by transferred T cells (Hollenbaugh et al., 2004; Hollenbaugh and Dutton, 2006). IFNγR1 and inducible nitric oxide synthase-2 (NOS2) expression in recipient mice were also required for tumor regression which indicated that CTL-expressed-IFNγ targeted host cells to eliminate skin tumors (Hollenbaugh et al., 2004; Hollenbaugh and Dutton, 2006). We recently demonstrated that transferred OT-I CTL induced F4/80+ macrophages within E.G7-OVA skin tumors to express tumoricidal concentrations of nitric oxide (NO) (Vicetti Miguel et al., 2010). Hence CD8+ T cell elimination of established E.G7-OVA skin tumors was indirect via their induction of tumoricidal activity in intratumoral macrophages. In contrast, established intraocular E.G7-OVA tumors were resistant to OT-I CTL transfer therapy although CTL infiltrated primary intraocular tumors and expressed IFNγ which induced macrophages to express NOS2 protein; the enzyme responsible for NO production. However, ocular tumor associated macrophages did not produce appreciable amounts of NO and were not tumoricidal (Vicetti Miguel et al., 2010). Therefore, factors within the immune privileged eye normally inhibit NOS2 enzymatic activity in macrophages which contributes to ocular tumor progression. These data highlight that the interplay between CD8+ T cells and intratumoral macrophages is critical for elimination of intraocular tumors and represents a potential target of CD8+ Treg (Figure 6).

## Uveal melanoma

Immune suppressive mechanisms, which maintain ocular immune privilege, should theoretically decrease immunosurveillance of intraocular malignancies. However, the most common intraocular tumor, uveal melanoma (UM), is a rare malignancy. The frequency of UM is over 30 times lower than cutaneous melanoma (Singh and Topham, 2003; Garbe and Leiter, 2009). One explanation for this paradox is that the expression of death inducing molecules within the eye limits tumor outgrowth. For example, tumor necrosis factor (TNF) related apoptosis inducing ligand (TRAIL) is expressed by tissues lining the interior of the eye (Lee et al., 2002) and targets transformed cells for apoptosis (Wiley et al., 1995). P815 tumor cells transduced to express TRAIL receptor DR5 failed to develop into ocular tumors when injected into the a.c. of the eye of Balb/C mice (Lee et al., 2002) which supports this notion. Therefore, the lower frequency of ocular malignancies may be due to augmented expression of death inducing molecules that induce apoptosis in transformed cells. However, when transformed cells become resistant to apoptosis several observations suggest that ocular immune privilege favors tumor growth and persistence.

Uveal melanoma is unique in that primary tumors with an “inflammatory phenotype,” characterized by significant infiltration with CD8+ T cells and CD68+ macrophages, are generally larger more vascularized tumors which express a genetic profile indicative of greater risk of liver metastasis (Maat et al., 2008). These data suggest that tumor-specific ocular immune responses are somehow converted within the ocular tumor microenvironment to favor tumor growth which is consistent with immunosuppression by ocular immune privilege.

CD8+ T cell function may be impaired within primary uveal melanomas as reduced CD3zeta chain expression, a marker of T cell dysfunction, was observed in T cells infiltrating ocular tumors that ultimately metastasized to the liver (Staibano et al., 2006). In addition, T cells isolated from primary uveal melanomas were generally non-responsive, proliferating poorly after stimulation (Ksander et al., 1998). In other malignancies reduced CD3zeta chain expression correlated with increased frequencies of activated CD11b+ CD15+ granulocytes in the blood (Schmielau and Finn, 2001; Zea et al., 2005) and we recently showed that CD11b+ CD15+ granulocytes were increased in a cohort of patients with primary UMs (McKenna et al., 2009) suggesting a possible mechanism for CD3zeta chain downmodulation. Although CD8+ T cells within primary UM appear to proliferate poorly, they still accumulate within primary uveal melanomas and indirect evidence suggests that they produce IFNγ. For example, primary UMs with increased numbers of CD8+ T cells are associated with increased HLA expression (de Waard-Siebinga et al., 1996) which is known to be influenced by IFNγ (de Waard-Siebinga et al., 1995). Moreover, in the E.G7-OVA/C57Bl/6 mouse model of intraocular tumor development we observed that CD8+ T cells which infiltrated intraocular tumors expressed IFNγ at levels which were equivalent to those observed in CD8+ T cells that infiltrated skin tumors (Vicetti Miguel et al., 2010).

IFNγ may actually promote immune evasion by ocular tumors as primary uveal melanoma lines treated with IFNγ expressed PD-L1 which inhibited T cell function *in vitro* (Yang et al., 2008). Similarly, IFNγ rendered uveal melanoma cell lines resistant to lysis by CD8+ T cells (Hallermalm et al., 2008). In addition, IFNγ is required for expression of the suppressive activity of CD8+ Treg in ACAID (Cone et al., 2007; Paunicka et al., 2011). Therefore, another explanation for primary uveal melanoma growth despite infiltration by CD8+ T cells is that these CD8+ T cells are not tumoricidal effectors but rather immune suppressive Treg.

As previously described the transcription factor FoxP3 faithfully identifies naturally occurring CD4+ Treg in mice and is also expressed in some induced CD4+ and CD8+ Tregs (Shevach, 2009). Two studies recently evaluated FoxP3 expression on T cells infiltrating primary uveal melanomas (Lagouros et al., 2009; Mougiakakos et al., 2010). FoxP3 expression was observed only in CD4+ T cells and their numbers were generally very low and correlated with the size of tumors and frequency of CD3+ T cells (Lagouros et al., 2009). As both tumor size and CD3+ T infiltration are negative prognostic indicators (Damato et al., 2011), it is difficult to discern the influence of CD4+ FOXP3+ Treg on tumor progression. However, the presence of FOXP3+ Treg in cycloxygenase-2 (COX-2) positive tumors did predict poor survival of uveal melanoma patients (Mougiakakos et al., 2010). It is important to note that these studies could not exclude that infiltrating CD8+ T cells, though FoxP3 negative, were immunosuppressive Treg.

Macrophages have also been shown to influence tumor growth (Mantovani et al., 2002). For example, macrophages stimulated with IFNγ and LPS, termed M1, demonstrate tumoricidal activity through expression of reactive oxygen and nitrogen production. In contrast, macrophages stimulated with IL-4 and IL-13, termed M2, are not tumoricidal and actually tumor promoting via expression of angiogenic factors including VEGF. Macrophages within primary uveal melanomas express CD163 which is a marker of M2 macrophages (Bronkhorst et al., 2010) and primary UMs heavily infiltrated with macrophages are more vascularized (Makitie et al., 2001). Taken together these data suggest that ocular tumor associated macrophages may promote tumor growth by inducing tumor angiogenesis. Therefore, it is possible that CD8+ Treg may contribute to ocular tumor growth by maintaining ocular tumor associated macrophages in an M2 phenotype.

## Conclusions

It is very clear that progressive growth of intraocular tumors generates CD8+ Treg that inhibit CD4+ T cell dependent DTH responses to tumor Ags. However, as restoration of CD4+ T cell responses to tumor Ags does not assure ocular tumor elimination the contribution of CD8+ Treg in ocular tumor progression remains unclear. Interestingly, rejection of certain tumors transplanted into the a.c. of the eye involves a destructive process resembling a “DTH-like” response that requires CD8+ T cells and IFNγ. As several observations indicate that T cell production of IFNγ is not impaired within ocular tumors, CD8+ Treg may target macrophages in progressively growing ocular tumors to prevent their expression of inflammatory mediators that promote DTH responses which destroy the tumor vasculature, and/or directly kill tumor cells (Figure 6). One challenge to defining the role of CD8+ Treg in ocular tumor progression is a specific marker that discerns CD8+ CTL effectors from CD8+ Tregs. While the selective expression of CD103 on *in vitro* generated CD8+ Treg is encouraging (Keino et al., 2006a), *in vivo* expression of CD103 by ACAID CD8+ Treg has not been shown. Future experimentation which compares and contrasts tumor-specific immune responses in splenectomized mice that do not generate CD8+ Treg and eliminate tumors placed in the a.c. to mice with progressively growing intraocular tumors should help to define potential targets for CD8+ Tregs and may identify novel molecules expressed by CD8+ Treg.

### Conflict of interest statement

The authors declare that the research was conducted in the absence of any commercial or financial relationships that could be construed as a potential conflict of interest.

## References

[B1] Abi-Hanna D. Wakefield D. Watkins S. (1988). HLA antigens in ocular tissues. I. *In vivo* expression in human eyes. Transplantation 45, 610–613 3347938 10.1097/00007890-198803000-00021

[B2] Apte R. S. Sinha D. Mayhew E. Wistow G. J. Niederkorn J. Y. (1998). Cutting edge: role of macrophage migration inhibitory factor in inhibiting NK cell activity and preserving immune privilege. J. Immunol. 160, 5693–5696 9637476

[B3] Ashour H. M. Niederkorn J. Y. (2006). Gammadelta T cells promote anterior chamber-associated immune deviation and immune privilege through their production of IL-10. J. Immunol. 177, 8331–8337 17142729 10.4049/jimmunol.177.12.8331

[B4] Benezra D. Sachs U. (1974). Growth factors in aqueous humor of normal and inflamed eyes of rabbits. Invest. Ophthalmol. 13, 868–870 4610416

[B5] Bill A. (1977). Basic physiology of the drainage of aqueous humor. Exp.Eye Res. 25(Suppl.) 291–304 412692 10.1016/s0014-4835(77)80025-0

[B6] Blankenstein T. (2005). The role of tumor stroma in the interaction between tumor and immune system. Curr. Opin. Immunol. 17, 180–186 10.1016/j.coi.2005.01.008 15766679

[B7] Boonman Z. F. Schurmans L. R. Van R. N. Melief C. J. Toes R. E. Jager M. J. (2006). Macrophages are vital in spontaneous intraocular tumor eradication. Invest. Ophthalmol. Vis. Sci. 47, 2959–2965 10.1167/iovs.05-1427 16799039

[B8] Bronkhorst I. H. Ly L. V. Jordanova E. S. Vrolijk H. Versluis M. Luyten G. P. Jager M. J. (2010). Detection of M2 macrophages in uveal melanoma and relation with survival. Invest. Ophthalmol. Vis. Sci. 52, 643–650 10.1167/iovs.10-5979 20811059

[B9] Cone R. E. Chattopadhyay S. Sharafieh R. Lemire Y. O'Rourke J. (2009a). The suppression of hypersensitivity by ocular-induced CD8(+) T cells requires compatibility in the Qa-1 haplotype. Immunol. Cell Biol. 87, 241–248 10.1038/icb.2008.102 19139762 PMC2658723

[B10] Cone R. E. Chattopadhyay S. Sharafieh R. Lemire Y. O'Rourke J. Flavell R. A. Clark R. B. (2009b). T cell sensitivity to TGF-beta is required for the effector function but not the generation of splenic CD8+ regulatory T cells induced via the injection of antigen into the anterior chamber. Int. Immunol. 21, 567–574 10.1093/intimm/dxp023 19325036 PMC2675031

[B11] Cone R. E. Li X. Sharafieh R. O'Rourke J. Vella A. T. (2007). The suppression of delayed-type hypersensitivity by CD8+ regulatory T cells requires interferon-gamma. Immunology 120, 112–119 10.1111/j.1365-2567.2006.02486.x 17052246 PMC2265875

[B12] Coursey T. G. Chen P. W. Niederkorn J. Y. (2011). Abrogating TNF-alpha expression prevents bystander destruction of normal tissues during iNOS-mediated elimination of intraocular tumors. Cancer Res. 71, 2445–2454 10.1158/0008-5472.CAN-10-2628 21307132 PMC3108159

[B13] Cousins S. W. McCabe M. M. Danielpour D. Streilein J. W. (1991). Identification of transforming growth factor-beta as an immunosuppressive factor in aqueous humor. Invest. Ophthalmol. Vis. Sci. 32, 2201–2211 2071334

[B14] Crane I. J. Liversidge J. (2008). Mechanisms of leukocyte migration across the blood-retina barrier. Semin. Immunopathol. 30, 165–177 10.1007/s00281-008-0106-7 18305941 PMC2315689

[B15] Dace D. S. Chen P. W. Alizadeh H. Niederkorn J. Y. (2007). Ocular immune privilege is circumvented by CD4+ T cells, leading to the rejection of intraocular tumors in an IFN-{gamma}-dependent manner. J. Leukoc. Biol. 81, 421–429 10.1189/jlb.0806489 17077163

[B16] Dace D. S. Chen P. W. Niederkorn J. Y. (2008). CD4+ T-cell-dependent tumour rejection in an immune-privileged environment requires macrophages. Immunology 123, 367–377 10.1111/j.1365-2567.2007.02700.x 17944931 PMC2433326

[B17] Damato B. Eleuteri A. Taktak A. F. Coupland S. E. (2011). Estimating prognosis for survival after treatment of choroidal melanoma. Prog. Retin. Eye Res. 30, 285–295 10.1016/j.preteyeres.2011.05.003 21658465

[B18] de la Cruz P. O. Jr. Specht C. S. McLean I. W. (1990). Lymphocytic infiltration in uveal malignant melanoma. Cancer 65, 112–115 2293857 10.1002/1097-0142(19900101)65:1<112::aid-cncr2820650123>3.0.co;2-x

[B19] de Waard-Siebinga I. Creyghton W. M. Kool J. Jager M. J. (1995). Effects of interferon alfa and gamma on human uveal melanoma cells *in vitro*. Br. J. Ophthalmol. 79, 847–855 7488605 10.1136/bjo.79.9.847 PMC505272

[B20] de Waard-Siebinga I. Hilders C. G. Hansen B. E. van Delft J. L. Jager M. J. (1996). HLA expression and tumor-infiltrating immune cells in uveal melanoma. Graefes Arch. Clin. Exp. Ophthalmol. 234, 34–42 8750848 10.1007/BF00186516

[B21] D'Orazio T. J. Mayhew E. Niederkorn J. Y. (2001). Ocular immune privilege promoted by the presentation of peptide on tolerogenic B cells in the spleen. II. Evidence for presentation by Qa-1. J. Immunol. 166, 26–32 11123273 10.4049/jimmunol.166.1.26

[B22] D'Orazio T. J. Niederkorn J. Y. (1998). A novel role for TGF-beta and IL-10 in the induction of immune privilege. J. Immunol. 160, 2089–2098 9498745

[B22a] Durie F. H. Campbell A. M. Lee W. R. Damato B. (1990). Analysis of lymphocytic infiltration in uveal melanoma. Invest. Ophthalmol. Vis. Sci. 31, 2106–2110 2211008

[B23] Egan R. M. Yorkey C. Black R. Loh W. K. Stevens J. L. Woodward J. G. (1996). Peptide-specific T cell clonal expansion *in vivo* following immunization in the eye, an immune-privileged site. J. Immunol. 157, 2262–2271 8805623

[B24] Faunce D. E. Sonoda K. H. Stein-Streilein J. (2001). MIP-2 recruits NKT cells to the spleen during tolerance induction. J. Immunol. 166, 313–321 11123307 10.4049/jimmunol.166.1.313

[B25] Faunce D. E. Stein-Streilein J. (2002). NKT cell-derived RANTES recruits APCs and CD8+ T cells to the spleen during the generation of regulatory T cells in tolerance. J. Immunol. 169, 31–38 12077225 10.4049/jimmunol.169.1.31

[B26] Ferguson T. A. Hayashi J. D. Kaplan H. J. (1989). The immune response and the eye. III. Anterior chamber-associated immune deviation can be adoptively transferred by serum. J. Immunol. 143, 821–826 2473113

[B27] Fontenot J. D. Gavin M. A. Rudensky A. Y. (2003). Foxp3 programs the development and function of CD4+CD25+ regulatory T cells. Nat. Immunol. 4, 330–336 10.1038/ni904 12612578

[B28] Garbe C. Leiter U. (2009). Melanoma epidemiology and trends. Clin. Dermatol. 27, 3–9 10.1016/j.clindermatol.2008.09.001 19095149

[B29] Gershon R. K. Kondo K. (1971). Infectious immunological tolerance. Immunology 21, 903–914 4943147 PMC1408252

[B30] Griffith T. S. Brincks E. L. Gurung P. Kucaba T. A. Ferguson T. A. (2011). Systemic immunological tolerance to ocular antigens is mediated by TRAIL-expressing CD8+ T cells. J. Immunol. 186, 791–798 10.4049/jimmunol.1002678 21169546 PMC3075597

[B31] Griffith T. S. Brunner T. Fletcher S. M. Green D. R. Ferguson T. A. (1995a). Fas ligand-induced apoptosis as a mechanism of immune privilege. Science 270, 1189–1192 10.1126/science.270.5239.1189 7502042

[B32] Griffith T. S. Herndon J. M. Lima J. Kahn M. Ferguson T. A. (1995b). The immune response and the eye. TCR alpha-chain related molecules regulate the systemic immunity to antigen presented in the eye. Int. Immunol. 7, 1617–1625 8562507 10.1093/intimm/7.10.1617

[B33] Griffith T. S. Yu X. Herndon J. M. Green D. R. Ferguson T. A. (1996). CD95-induced apoptosis of lymphocytes in an immune privileged site induces immunological tolerance. Immunity 5, 7–16 10.1016/S1074-7613(00)80305-2 8758890

[B34] Hadjikouti C. A. Wang Y. O'Rourke J. Cone R. E. (1995). Intracameral injection of antigen potentiates the production of antigen-specific T cell proteins in serum after the induction of delayed-type hypersensitivity. Invest. Ophthalmol. Vis. Sci. 36, 1470–1476 7775125

[B35] Hallermalm K. Seki K. De G. A. Motyka B. Bleackley R. C. Jager M. J. Froelich C. J. Kiessling R. Levitsky V. Levitskaya J. (2008). Modulation of the tumor cell phenotype by IFN-gamma results in resistance of uveal melanoma cells to granule-mediated lysis by cytotoxic lymphocytes. J. Immunol. 180, 3766–3774 18322182 10.4049/jimmunol.180.6.3766

[B36] Hollenbaugh J. A. Dutton R. W. (2006). IFN-gamma regulates donor CD8 T cell expansion, migration, and leads to apoptosis of cells of a solid tumor. J. Immunol. 177, 3004–3011 16920936 10.4049/jimmunol.177.5.3004

[B37] Hollenbaugh J. A. Reome J. Dobrzanski M. Dutton R. W. (2004). The rate of the CD8-dependent initial reduction in tumor volume is not limited by contact-dependent perforin, Fas ligand, or TNF-mediated cytolysis. J. Immunol. 173, 1738–1743 15265903 10.4049/jimmunol.173.3.1738

[B38] Hori J. Wang M. Miyashita M. Tanemoto K. Takahashi H. Takemori T. Okumura K. Yagita H. Azuma M. (2006). B7-H1-induced apoptosis as a mechanism of immune privilege of corneal allografts. J. Immunol. 177, 5928–5935 17056517 10.4049/jimmunol.177.9.5928

[B39] Hu D. Ikizawa K. Lu L. Sanchirico M. E. Shinohara M. L. Cantor H. (2004). Analysis of regulatory CD8 T cells in Qa-1-deficient mice. Nat. Immunol. 5, 516–523 10.1038/ni1063 15098030

[B40] Jiang L. He H. Yang P. Lin X. Zhou H. Huang X. Kijlstra A. (2009). Splenic CD8+ T cells secrete TGF-beta1 to exert suppression in mice with anterior chamber-associated immune deviation. Graefes Arch. Clin. Exp. Ophthalmol. 247, 87–92 10.1007/s00417-008-0947-8 18797912

[B41] Kaiser C. J. Ksander B. R. Streilein J. W. (1989). Inhibition of lymphocyte proliferation by aqueous humor. Reg. Immunol. 2, 42–49 2534948

[B42] Kaplan H. J. Streilein J. W. (1977). Immune response to immunization via the anterior chamber of the eye. I. F1-lymphocyte induced immune deviation. J. Immunol. 118, 809–814 321682

[B43] Kapp J. A. Honjo K. Kapp L. M. Goldsmith K. Bucy R. P. (2007). Antigen, in the presence of TGF-beta, induces up-regulation of FoxP3gfp+ in CD4+ TCR transgenic T cells that mediate linked suppression of CD8+ T cell responses. J. Immunol. 179, 2105–2114 17675469 10.4049/jimmunol.179.4.2105

[B44] Kapp J. A. Honjo K. Kapp L. M. Xu X. Cozier A. Bucy R. P. (2006). TCR transgenic CD8+ T cells activated in the presence of TGFbeta express FoxP3 and mediate linked suppression of primary immune responses and cardiac allograft rejection. Int. Immunol. 18, 1549–1562 10.1093/intimm/dxl088 16966495

[B45] Keino H. Masli S. Sasaki S. Streilein J. W. Stein-Streilein J. (2006a). CD8+ T regulatory cells use a novel genetic program that includes CD103 to suppress Th1 immunity in eye-derived tolerance. Invest. Ophthalmol. Vis. Sci. 47, 1533–1542 10.1167/iovs.04-1454 16565389

[B46] Keino H. Takeuchi M. Kezuka T. Hattori T. Usui M. Taguchi O. Streilein J. W. Stein-Streilein J. (2006b). Induction of eye-derived tolerance does not depend on naturally occurring CD4+CD25+ T regulatory cells. Invest. Ophthalmol. Vis. Sci. 47, 1047–1055 10.1167/iovs.05-0110 16505040

[B47] Kezuka T. Streilein J. W. (2000). *In vitro* generation of regulatory CD8+ T cells similar to those found in mice with anterior chamber-associated immune deviation. Invest. Ophthalmol. Vis. Sci. 41, 1803–1811 10845601

[B48] Knisely T. L. Luckenbach M. W. Fischer B. J. Niederkorn J. Y. (1987). Destructive and nondestructive patterns of immune rejection of syngeneic intraocular tumors. J. Immunol. 138, 4515–4523 3108394

[B49] Kosiewicz M. M. Alard P. Liang S. Clark S. L. (2004). Mechanisms of tolerance induced by transforming growth factor-beta-treated antigen-presenting cells: CD8 regulatory T cells inhibit the effector phase of the immune response in primed mice through a mechanism involving Fas ligand. Int. Immunol. 16, 697–706 10.1093/intimm/dxh067 15096489

[B50] Kosiewicz M. M. Streilein J. W. (1996). Intraocular injection of class II-restricted peptide induces an unexpected population of CD8 regulatory cells. J. Immunol. 157, 1905–1912 8757308

[B51] Ksander B. R. Bando Y. Acevedo J. Streilein J. W. (1991). Infiltration and accumulation of precursor cytotoxic T-cells increase with time in progressively growing ocular tumors. Cancer Res. 51, 3153–3158 1904003

[B52] Ksander B. R. Geer D. C. Chen P. W. Salgaller M. L. Rubsamen P. Murray T. G. (1998). Uveal melanomas contain antigenically specific and non-specific infiltrating lymphocytes. Curr. Eye Res. 17, 165–173 9523095 10.1076/ceyr.17.2.165.5607

[B53] Ksander B. R. Hendricks R. L. (1987). Cell-mediated immune tolerance to HSV-1 antigens associated with reduced susceptibility to HSV-1 corneal lesions. Invest. Ophthalmol. Vis. Sci. 28, 1986–1993 2824400

[B54] Ksander B. R. Streilein J. W. (1989). Analysis of cytotoxic T cell responses to intracameral allogeneic tumors. Invest. Ophthalmol. Vis. Sci. 30, 323–329 2492486

[B55] Lagouros E. Salomao D. Thorland E. Hodge D. O. Vile R. Pulido J. S. (2009). Infiltrative T regulatory cells in enucleated uveal melanomas. Trans. Am. Ophthalmol. Soc. 107, 223–228 20126498 PMC2814577

[B56] Lai J. C. Lobanoff M. C. Fukushima A. Wawrousek E. F. Chan C. C. Whitcup S. M. Gery I. (1999). Uveitis induced by lymphocytes sensitized against a transgenically expressed lens protein. Invest. Ophthalmol. Vis. Sci. 40, 2735–2739 10509672

[B57] Lee H. O. Herndon J. M. Barreiro R. Griffith T. S. Ferguson T. A. (2002). TRAIL: a mechanism of tumor surveillance in an immune privileged site. J. Immunol. 169, 4739–4744 12391182 10.4049/jimmunol.169.9.4739

[B58] Li X. Taylor S. Zegarelli B. Shen S. O'Rourke J. Cone R. E. (2004). The induction of splenic suppressor T cells through an immune-privileged site requires an intact sympathetic nervous system. J. Neuroimmunol. 153, 40–49 10.1016/j.jneuroim.2004.04.008 15265662

[B59] Lin H. H. Faunce D. E. Stacey M. Terajewicz A. Nakamura T. Zhang-Hoover J. Kerley M. Mucenski M. L. Gordon S. Stein-Streilein J. (2005). The macrophage F4/80 receptor is required for the induction of antigen-specific efferent regulatory T cells in peripheral tolerance. J. Exp. Med. 201, 1615–1625 10.1084/jem.20042307 15883173 PMC2212925

[B60] Maat W. Ly L. V. Jordanova E. S. de Wolff-Rouendaal D. Schalij-Delfos N. E. Jager M. J. (2008). Monosomy of chromosome 3 and an inflammatory phenotype occur together in uveal melanoma. Invest. Ophthalmol. Vis. Sci. 49, 505–510 10.1167/iovs.07-0786 18234992

[B61] Makitie T. Summanen P. Tarkkanen A. Kivela T. (2001). Tumor-infiltrating macrophages (CD68(+) cells) and prognosis in malignant uveal melanoma. Invest. Ophthalmol. Vis. Sci. 42, 1414–1421 11381040

[B62] Mantovani A. Sozzani S. Locati M. Allavena P. Sica A. (2002). Macrophage polarization: tumor-associated macrophages as a paradigm for polarized M2 mononuclear phagocytes. Trends Immunol. 23, 549–555 10.1016/S1471-4906(02)02302-5 12401408

[B63] Masli S. Turpie B. Streilein J. W. (2006). Thrombospondin orchestrates the tolerance-promoting properties of TGFbeta-treated antigen-presenting cells. Int. Immunol. 18, 689–699 10.1093/intimm/dxl006 16569680

[B64] McKenna K. C. Anderson K. M. Kapp J. A. (2005). CD8+ T-cell tolerance induced by delivery of antigen to the anterior chamber is not the result of de facto intravenous or mucosal administration of antigen. Ocul. Immunol. Inflamm. 13, 149–157 10.1080/09273940590933520 16019674

[B65] McKenna K. C. Beatty K. M. Bilonick R. A. Schoenfield L. Lathrop K. L. Singh A. D. (2009). Activated CD11b+ CD15+ granulocytes increase in blood of uveal melanoma patients. Invest. Ophthalmol. Vis. Sci. 50, 4295–4303 10.1167/iovs.08-3012 19369244 PMC5333486

[B66] McKenna K. C. Kapp J. A. (2006). Accumulation of immunosuppressive CD11b+ myeloid cells correlates with the failure to prevent tumor growth in the anterior chamber of the eye. J. Immunol. 177, 1599–1608 16849468 10.4049/jimmunol.177.3.1599

[B67] McKenna K. C. Xu Y. Kapp J. A. (2002). Injection of soluble antigen into the anterior chamber of the eye induces expansion and functional unresponsiveness of antigen-specific CD8+ T cells. J. Immunol. 169, 5630–5637 12421942 10.4049/jimmunol.169.10.5630

[B68] Medawar P. (1948). Immunity to homologous grafted skin: III. The fate of skin homografts transplanted to the brain, to subcutaneous tissue, and to the anterior chamber of the eye. Br. J. Exp. Pathol. 29, 58–69 18865105 PMC2073079

[B69] Meecham W. J. Char D. H. Kaleta-Michaels S. (1992). Infiltrating lymphocytes and antigen expression in uveal melanoma. Ophthalmic Res. 24, 20–26 1608588 10.1159/000267140

[B70] Mougiakakos D. Johansson C. C. Trocme E. ll-Ericsson C. Economou M. A. Larsson O. Seregard S. Kiessling R. (2010). Intratumoral forkhead box P3-positive regulatory T cells predict poor survival in cyclooxygenase-2-positive uveal melanoma. Cancer 116, 2224–2233 10.1002/cncr.24999 20209608

[B71] Nakamura T. Sonoda K. H. Faunce D. E. Gumperz J. Yamamura T. Miyake S. Stein-Streilein J. (2003). CD4(+) NKT cells, but not conventional CD4(+) T cells, are required to generate efferent CD8(+) T regulatory cells following antigen inoculation in an immune-privileged site. J. Immunol. 171, 1266–1271 12874214 10.4049/jimmunol.171.3.1266

[B72] Niederkorn J. Y. (1984). Suppressed cellular immunity in mice harboring intraocular melanomas. Invest. Ophthalmol. Vis. Sci. 25, 447–454 6608506

[B73] Niederkorn J. Y. Benson J. L. Mayhew E. (1990). Efferent blockade of delayed-type hypersensitivity responses in the anterior chamber of the eye. Reg. Immunol. 3, 349–354 2132760

[B74] Niederkorn J. Y. Meunier P. C. (1985). Spontaneous immune rejection of intraocular tumors in mice. Invest. Ophthalmol. Vis. Sci. 26, 877–884 3924853

[B75] Niederkorn J. Y. Shadduck J. A. Streilein J. W. (1981). Immunogenetic basis for immunologic privilege in the anterior chamber of the eye. Immunogenetics 13, 227–236 6792066 10.1007/BF00350789

[B76] Niederkorn J. Y. Streilein J. W. (1982a). Analysis of antibody production induced by allogeneic tumor cells inoculated into the anterior chamber of the eye. Transplantation 33, 573–577 6808721 10.1097/00007890-198206000-00001

[B77] Niederkorn J. Y. Streilein J. W. (1982b). Induction of anterior chamber-associated immune deviation (ACAID) by allogeneic intraocular tumors does not require splenic metastases. J. Immunol. 128, 2470–2474 6804562

[B78] Niederkorn J. Y. Streilein J. W. (1983a). Alloantigens placed into the anterior chamber of the eye induce specific suppression of delayed-type hypersensitivity but normal cytotoxic T lymphocyte and helper T lymphocyte responses. J. Immunol. 131, 2670–2674 6196396

[B79] Niederkorn J. Y. Streilein J. W. (1983b). Intracamerally induced concomitant immunity: mice harboring progressively growing intraocular tumors are immune to spontaneous metastases and secondary tumor challenge. J. Immunol. 131, 2587–2594 6415174

[B80] Niederkorn J. Y. Streilein J. W. (1984). Adoptive transfer of immunity to intraocular tumors in mice. Invest. Ophthalmol. Vis. Sci. 25, 336–342 6421767

[B81] Nishida T. Taylor A. W. (1999). Specific aqueous humor factors induce activation of regulatory T cells. Invest. Ophthalmol. Vis. Sci. 40, 2268–2274 10476792

[B82] Patel S. P. Dana R. (2009). Corneal lymphangiogenesis: implications in immunity. Semin. Ophthalmol. 24, 135–138 10.1080/08820530902801320 19437348

[B83] Paunicka K. Chen P. W. Niederkorn J. Y. (2011). Role of IFN-gamma in the establishment of anterior chamber-associated immune deviation (ACAID)-induced CD8+ T regulatory cells. J. Leukoc. Biol. 91, 475–483 10.1189/jlb.0311173 22180630 PMC3289396

[B84] Perez V. L. Biuckians A. Streilein J. W. (2000). *In-vivo* impaired T helper 1 cell development in submandibular lymph nodes due to Il-12 deficiency following antigen injection into the anterior chamber of the eye. Ocul. Immunol. Inflamm. 8, 9–24 10806431

[B85] Qin Z. Schwartzkopff J. Pradera F. Kammertoens T. Seliger B. Pircher H. Blankenstein T. (2003). A critical requirement of interferon gamma-mediated angiostasis for tumor rejection by CD8+ T cells. Cancer Res. 63, 4095–4100 12874012

[B86] Radosevich M. Jager M. Ono S. J. (2007). Inhibition of MHC class II gene expression in uveal melanoma cells is due to methylation of the CIITA gene or an upstream activator. Exp. Mol. Pathol. 82, 68–76 10.1016/j.yexmp.2006.03.005 16650406

[B87] Radosevich M. Song Z. Gorga J. C. Ksander B. Ono S. J. (2004). Epigenetic silencing of the CIITA gene and posttranscriptional regulation of class II MHC genes in ocular melanoma cells. Invest. Ophthalmol. Vis. Sci. 45, 3185–3195 10.1167/iovs.04-0111 15326139

[B88] Rifa'i M. Kawamoto Y. Nakashima I. Suzuki H. (2004). Essential roles of CD8+CD122+ regulatory T cells in the maintenance of T cell homeostasis. J. Exp. Med. 200, 1123–1134 10.1084/jem.20040395 15520244 PMC2211869

[B89] Rosenbaum J. T. Howes E. L. Jr. English D. (1985). Ascorbate in aqueous humor protects against myeloperoxidase-induced oxidation. Am. J. Pathol. 120, 244–247 2992283 PMC1887821

[B90] Sakaguchi S. Sakaguchi N. Asano M. Itoh M. Toda M. (1995). Immunologic self-tolerance maintained by activated T cells expressing IL-2 receptor alpha-chains (CD25). Breakdown of a single mechanism of self-tolerance causes various autoimmune diseases. J. Immunol. 155, 1151–1164 7636184

[B91] Schmielau J. Finn O. J. (2001). Activated granulocytes and granulocyte-derived hydrogen peroxide are the underlying mechanism of suppression of t-cell function in advanced cancer patients. Cancer Res. 61, 4756–4760 11406548

[B92] Schuler T. Blankenstein T. (2003). Cutting edge: CD8+ effector T cells reject tumors by direct antigen recognition but indirect action on host cells. J. Immunol. 170, 4427–4431 12707316 10.4049/jimmunol.170.9.4427

[B93] Schurmans L. R. Diehl L. den Boer A. T. Sutmuller R. P. Boonman Z. F. Medema J. P. van der Voort E. I. Laman J. Melief C. J. Jager M. J. Toes R. E. (2001). Rejection of intraocular tumors by CD4(+) T cells without induction of phthisis. J. Immunol. 167, 5832–5837 11698457 10.4049/jimmunol.167.10.5832

[B94] Shevach E. M. (2009). Mechanisms of foxp3+ T regulatory cell-mediated suppression. Immunity 30, 636–645 10.1016/j.immuni.2009.04.010 19464986

[B95] Simon M. M. Hausmann M. Tran T. Ebnet K. Tschopp J. ThaHla R. Mullbacher A. (1997). *In vitro*- and *ex vivo*-derived cytolytic leukocytes from granzyme A x B double knockout mice are defective in granule-mediated apoptosis but not lysis of targeT cells. J. Exp. Med. 186, 1781–1786 10.1084/jem.186.10.1781 9362539 PMC2199142

[B96] Singh A. D. Topham A. (2003). Incidence of uveal melanoma in the United States: 1973–1997. Ophthalmology 110, 956–961 10.1016/S0161-6420(03)00078-2 12750097

[B97] Skelsey M. E. Mellon J. Niederkorn J. Y. (2001). Gamma delta T cells are needed for ocular immune privilege and corneal graft survival. J. Immunol. 166, 4327–4333 11254685 10.4049/jimmunol.166.7.4327

[B98] Sonoda K. H. Exley M. Snapper S. Balk S. P. Stein-Streilein J. (1999). CD1-reactive natural killer T cells are required for development of systemic tolerance through an immune-privileged site. J. Exp. Med. 190, 1215–1226 10.1084/jem.190.9.1215 10544194 PMC2195676

[B99] Sonoda K. H. Faunce D. E. Taniguchi M. Exley M. Balk S. Stein-Streilein J. (2001). NK T cell-derived IL-10 is essential for the differentiation of antigen-specific T regulatory cells in systemic tolerance. J. Immunol. 166, 42–50 11123275 10.4049/jimmunol.166.1.42

[B100] Sonoda K. H. Stein-Streilein J. (2002). CD1d on antigen-transporting APC and splenic marginal zone B cells promotes NKT cell-dependent tolerance. Eur. J. Immunol. 32, 848–857 10.1002/1521-4141(200203)32:3<848::AID-IMMU848>3.0.CO;2-I 11870629

[B101] Staibano S. Mascolo M. Tranfa F. Salvatore G. Mignogna C. Bufo P. Nugnes L. Bonavolonta G. De R. G. (2006). Tumor infiltrating lymphocytes in uveal melanoma: a link with clinical behavior? Int. J. Immunopathol. Pharmacol. 19, 171–179 16569355

[B102] Streilein J. W. Niederkorn J. Y. (1981). Induction of anterior chamber-associated immune deviation requires an intact, functional spleen. J. Exp. Med. 153, 1058–1067 6788883 10.1084/jem.153.5.1058 PMC2186172

[B103] Streilein J. W. Niederkorn J. Y. (1985). Characterization of the suppressor cell(s) responsible for anterior chamber-associated immune deviation (ACAID) induced in BALB/c mice by P815 cells. J. Immunol. 134, 1381–1387 3155766

[B104] Stuart P. M. Griffith T. S. Usui N. Pepose J. Yu X. Ferguson T. A. (1997). CD95 ligand (FasL)-induced apoptosis is necessary for corneal allograft survival. J. Clin. Invest. 99, 396–402 10.1172/JCI119173 9022072 PMC507812

[B105] Sugita S. Horie S. Nakamura O. Futagami Y. Takase H. Keino H. Aburatani H. Katunuma N. Ishidoh K. Yamamoto Y. Mochizuki M. (2008). Retinal pigment epithelium-derived CTLA-2alpha induces TGFbeta-producing T regulatory cells. J. Immunol. 181, 7525–7536 19017942 10.4049/jimmunol.181.11.7525

[B106] Sugita S. Horie S. Nakamura O. Maruyama K. Takase H. Usui Y. Takeuchi M. Ishidoh K. Koike M. Uchiyama Y. Peters C. Yamamoto Y. Mochizuki M. (2009). Acquisition of T regulatory function in cathepsin L-inhibited T cells by eye-derived CTLA-2alpha during inflammatory conditions. J. Immunol. 183, 5013–5022 10.4049/jimmunol.0901623 19801522

[B107] Sugita S. Horie S. Yamada Y. Keino H. Usui Y. Takeuchi M. Mochizuki M. (2010). Suppression of bystander T helper 1 cells by iris pigment epithelium-inducing regulatory T cells via negative costimulatory signals. Invest. Ophthalmol. Vis. Sci. 51, 2529–2536 10.1167/iovs.09-4460 19959639

[B108] Sugita S. Ng T. F. Lucas P. J. Gress R. E. Streilein J. W. (2006). B7+ iris pigment epithelium induce CD8+ T regulatory cells; both suppress CTLA-4+ T cells. J. Immunol. 176, 118–127 16365402 10.4049/jimmunol.176.1.118

[B109] Sugita S. Yamada Y. Horie S. Nakamura O. Ishidoh K. Yamamoto Y. Yamagami S. Mochizuki M. (2011). Induction of T regulatory cells by cytotoxic T-lymphocyte antigen-2alpha on corneal endothelial cells. Invest. Ophthalmol. Vis. Sci. 52, 2598–2605 10.1167/iovs.10-6322 21245393

[B110] Takeuchi M. Alard P. Streilein J. W. (1998). TGF-b promotes immune deviation by altering accessory signals of antigen-presenting cells. J. Immunol. 160, 1589–1597 9469414

[B111] Taylor A. W. Alard P. Yee D. G. Streilein J. W. (1997). Aqueous humor induces transforming growth factor-beta (TGF-beta)-producing regulatory T-cells. Curr. Eye Res. 16, 900–908 9288451 10.1076/ceyr.16.9.900.5043

[B112] Taylor A. W. Streilein J. W. Cousins S. W. (1994). Immunoreactive vasoactive intestinal peptide contributes to the immunosuppressive activity of normal aqueous humor. J. Immunol. 153, 1080–1086 8027541

[B113] Taylor A. W. Yee D. G. (2003). Somatostatin is an immunosuppressive factor in aqueous humor. Invest. Ophthalmol. Vis. Sci. 44, 2644–2649 10.1167/iovs.02-1216 12766068

[B114] Tennakoon D. K. Mehta R. S. Ortega S. B. Bhoj V. Racke M. K. Karandikar N. J. (2006). Therapeutic induction of regulatory, cytotoxic CD8+ T cells in multiple sclerosis. J. Immunol. 176, 7119–7129 16709875 10.4049/jimmunol.176.11.7119

[B115] Vega J. L. Keino H. Masli S. (2009). Surgical denervation of ocular sympathetic afferents decreases local transforming growth factor-beta and abolishes immune privilege. Am. J. Pathol. 175, 1218–1225 10.2353/ajpath.2009.090264 19700755 PMC2731140

[B116] Vicetti Miguel R. D. Cherpes T. L. Watson L. J. McKenna K. C. (2010). CTL induction of tumoricidal nitric oxide production by intratumoral macrophages is critical for tumor elimination. J. Immunol. 185, 6706–6718 10.4049/jimmunol.0903411 21041723 PMC3152256

[B117] Vlad G. D'Agati V. D. Zhang Q. Y. Liu Z. Ho E. K. Mohanakumar T. Hardy M. A. Cortesini R. Suciu-Foca N. (2008). Immunoglobulin-like transcript 3-Fc suppresses T-cell responses to allogeneic human islet transplants in hu-NOD/SCID mice. Diabetes 57, 1878–1886 10.2337/db08-0054 18420485 PMC2453624

[B118] Waldrep J. C. Kaplan H. J. (1983). Anterior chamber associated immune deviation induced by TNP-splenocytes (TNP-ACAID). I. Systemic tolerance mediated by suppressor T-cells. Invest. Ophthalmol. Vis. Sci. 24, 1086–1092 6192107

[B119] Wall L. Burke F. Barton C. Smyth J. Balkwill F. (2003). IFN-gamma induces apoptosis in ovarian cancer cells *in vivo* and *in vitro*. Clin. Cancer Res. 9, 2487–2496 12855622

[B120] Wang S. Boonman Z. F. Li H. C. He Y. Jager M. J. Toes R. E. Niederkorn J. Y. (2003). Role of TRAIL and IFN-gamma in CD4+ T cell-dependent tumor rejection in the anterior chamber of the eye. J. Immunol. 171, 2789–2796 12960299 10.4049/jimmunol.171.6.2789

[B121] Wang Y. Goldschneider I. Foss D. Wu D. Y. O'Rourke J. Cone R. E. (1997). Direct thymic involvement in anterior chamber-associated immune deviation: evidence for a nondeletional mechanism of centrally induced tolerance to extrathymic antigens in adult mice. J. Immunol. 158, 2150–2155 9036960

[B122] Wang Y. Goldschneider I. O'Rourke J. Cone R. E. (2001). Blood mononuclear cells induce regulatory NK T thymocytes in anterior chamber-associated immune deviation. J. Leukoc. Biol. 69, 741–746 11358982

[B123] Wilbanks G. A. Mammolenti M. Streilein J. W. (1991). Studies on the induction of anterior chamber-associated immune deviation (ACAID). II. Eye-derived cells participate in generating blood-borne signals that induce ACAID. J. Immunol. 146, 3018–3024 2016537

[B124] Wilbanks G. A. Mammolenti M. Streilein J. W. (1992). Studies on the induction of anterior chamber-associated immune deviation (ACAID). III. Induction of ACAID depends upon intraocular transforming growth factor-b. Eur. J. Immunol. 22, 165–173 10.1002/eji.1830220125 1530916

[B125] Wilbanks G. A. Streilein J. W. (1991). Studies on the induction of anterior chamber-associated immune deviation (ACAID). 1. Evidence that an antigen-specific, ACAID-inducing, cell-associated signal exists in the peripheral blood. J. Immunol. 146, 2610–2617 1707912

[B126] Wilbanks G. A. Streilein J. W. (1992). Macrophages capable of inducing anterior chamber associated immune deviation demonstrate spleen-seeking migratory properties. Reg. Immunol. 4, 130–137 1303096

[B127] Wilbanks G. A. Streilein J. W. (1990). Characterization of suppressor cells in anterior chamber-associated immune deviation (ACAID) induced by soluble antigen. Evidence of two functionally and phenotypically distinct T-suppressor cell populations. Immunology 71, 383–389 1702748 PMC1384437

[B128] Wiley S. R. Schooley K. Smolak P. J. Din W. S. Huang C. P. Nicholl J. K. Sutherland G. R. Smith T. D. Rauch C. Smith C. A. (1995). Identification and characterization of a new member of the TNF family that induces apoptosis. Immunity 3, 673–682 8777713 10.1016/1074-7613(95)90057-8

[B129] Xu H. Manivannan A. Liversidge J. Sharp P. F. Forrester J. V. Crane I. J. (2003). Requirements for passage of T lymphocytes across non-inflamed retinal microvessels. J. Neuroimmunol. 142, 47–57 10.1016/S0165-5728(03)00258-3 14512163

[B130] Xu Y. Kapp J. A. (2002). Gamma delta T cells in anterior chamber-induced tolerance in CD8(+) CTL responses. Invest. Ophthalmol. Vis. Sci. 43, 3473–3479 12407158

[B131] Yamagami S. Dana M. R. (2001). The critical role of lymph nodes in corneal alloimmunization and graft rejection. Invest. Ophthalmol. Vis. Sci. 42, 1293–1298 11328742

[B132] Yamagami S. Kawashima H. Tsuru T. Yamagami H. Kayagaki N. Yagita H. Okumura K. Gregerson D. S. (1997). Role of Fas-Fas ligand interactions in the immunorejection of allogeneic mouse corneal transplants. Transplantation 64, 1107–1111 9355824 10.1097/00007890-199710270-00004

[B133] Yang W. Chen P. W. Li H. Alizadeh H. Niederkorn J. Y. (2008). PD-L1, PD-1 interaction contributes to the functional suppression of T-cell responses to human uveal melanoma cells *in vitro*. Invest. Ophthalmol. Vis. Sci. 49, 2518–2525 10.1167/iovs.07-1606 18296654 PMC2465808

[B134] Zea A. H. Rodriguez P. C. Atkins M. B. Hernandez C. Signoretti S. Zabaleta J. McDermott D. Quiceno D. Youmans A. O'Neill A. Mier J. Ochoa A. C. (2005). Arginase-producing myeloid suppressor cells in renal cell carcinoma patients: a mechanism of tumor evasion. Cancer Res. 65, 3044–3048 10.1158/0008-5472.CAN-04-4505 15833831

[B135] Zhou R. Horai R. Silver P. B. Mattapallil M. J. Zarate-Blades C. R. Chong W. P. Chen J. Rigden R. C. Villasmil R. Caspi R. R. (2012). The living eye “disarms” uncommitted autoreactive T cells by converting them to Foxp3(+) regulatory cells following local antigen recognition. J. Immunol. 188, 1742–1750 10.4049/jimmunol.1102415 22238462 PMC3273602

